# Self-Regulation of Anterior Insula with Real-Time fMRI and Its Behavioral Effects in Obsessive-Compulsive Disorder: A Feasibility Study

**DOI:** 10.1371/journal.pone.0135872

**Published:** 2015-08-24

**Authors:** Korhan Buyukturkoglu, Hans Roettgers, Jens Sommer, Mohit Rana, Leonie Dietzsch, Ezgi Belkis Arikan, Ralf Veit, Rahim Malekshahi, Tilo Kircher, Niels Birbaumer, Ranganatha Sitaram, Sergio Ruiz

**Affiliations:** 1 Graduate School of Neural & Behavioural Sciences, International Max Planck Research School, University of Tübingen, Tuebingen, Germany; 2 Institute for Medical Psychology and Behavioural Neurobiology, University of Tuebingen, Tuebingen, Germany; 3 Department of Psychiatry and Psychotherapy, Philipps-University Marburg, Marburg, Germany; 4 Ospedale San Camillo, Istituto di Ricovero e Cura a Carattere Scientifico, Venezia, Italy; 5 Department of Biomedical Engineering, University of Florida, Gainesville, Florida, United States of America; 6 Departamento de Psiquiatría, Escuela de Medicina, Centro Interdisciplinario de Neurociencia, Pontificia Universidad Católica de Chile, Santiago, Chile; University of Ariel, ISRAEL

## Abstract

**Introduction:**

Obsessive-compulsive disorder (OCD) is a common and chronic condition that can have disabling effects throughout the patient's lifespan. Frequent symptoms among OCD patients include fear of contamination and washing compulsions. Several studies have shown a link between contamination fears, disgust over-reactivity, and insula activation in OCD. In concordance with the role of insula in disgust processing, new neural models based on neuroimaging studies suggest that abnormally high activations of insula could be implicated in OCD psychopathology, at least in the subgroup of patients with contamination fears and washing compulsions.

**Methods:**

In the current study, we used a Brain Computer Interface (BCI) based on real-time functional magnetic resonance imaging (rtfMRI) to aid OCD patients to achieve down-regulation of the Blood Oxygenation Level Dependent (BOLD) signal in anterior insula. Our first aim was to investigate whether patients with contamination obsessions and washing compulsions can learn to volitionally decrease (down-regulate) activity in the insula in the presence of disgust/anxiety provoking stimuli. Our second aim was to evaluate the effect of down-regulation on clinical, behavioural and physiological changes pertaining to OCD symptoms. Hence, several pre- and post-training measures were performed, i.e., confronting the patient with a disgust/anxiety inducing real-world object (Ecological Disgust Test), and subjective rating and physiological responses (heart rate, skin conductance level) of disgust towards provoking pictures.

**Results:**

Results of this pilot study, performed in 3 patients (2 females), show that OCD patients can gain self-control of the BOLD activity of insula, albeit to different degrees. In two patients positive changes in behaviour in the EDT were observed following the rtfMRI trainings. Behavioural changes were also confirmed by reductions in the negative valence and in the subjective perception of disgust towards symptom provoking images.

**Conclusion:**

Although preliminary, results of this study confirmed that insula down-regulation is possible in patients suffering from OCD, and that volitional decreases of insula activation could be used for symptom alleviation in this disorder.

## Introduction

Obsessive-compulsive disorder (OCD) is among the most disabling anxiety conditions and accounts for more than half of serious anxiety cases [1]. It can have disabling effects throughout the patient's lifespan. To be diagnosed with OCD, a person must have obsessions and/or compulsions [2]. Among the most common obsessions are contamination fears (usually followed by washing compulsions), which are intense and intrusive feelings of being polluted or infected by contact with the dirty, infectious or soiled objects [3,4].

Prior work on the neural basis of OCD with neuroimaging techniques suggested an abnormal activation of several brain areas including the prefrontal cortex, orbitofrontal cortex, parietal cortex, cingulate gyrus, putamen, globus pallidus, nucleus accumbens and thalamus [5–7]. Hence, current models of the disease hypothesize a dysfunction in the above cortico-subcortical circuitry [8–10].

Pharmacological therapy (performed by drugs that exert their action on the serotoninergic neurotransmission), and cognitive behavioral therapy (CBT) are the most commonly used methods in the treatment of OCD [11–13]. Functional brain imaging (PET, SPECT, fMRI) studies exploring the brain activity of the OCD patients show decreased activity levels in several brain areas after successful treatment (with these and other therapeutic approuches), mainly in nucleus accumbens (NAc), anterior cingulate cortex (ACC), thalamus, orbitofrontal cortex (OFC) and insula in parallel with symptom improvement [14–20].

More recent efforts have tried to elucidate OCD’s neural basis in concordance with the heterogenic presentation of the disorder. In this sense, several studies have shown a link between contamination fears, disgust over-reactivity, and insula activation. Activation in the insula is associated with interoceptive/subjective feelings (e.g., pain, temperature, or itch stimuli), and the anterior part of this paralimbic structure is involved in disgust processing as a part of the gustatory cortex, containing neurons that respond to pleasant and unpleasant taste [21, 22].

Several sources of evidence support the aforementioned link between contamination fears, disgust over-reactivity, and insula activation. First, disgust is strongly associated with the fear of contamination and subsequent washing compulsions [4, 23, 24]. Furthermore, heightened disgust feelings towards disgust-inducing stimuli, and greater behavioral avoidance from disgusting objects, situations and places are commonly observed in OCD patients, especially those with contamination fears and washing compulsions [25–35]. On the other hand, findings from functional neuroimaging studies have indicated that when subjects are presented disgust inducing stimuli, the insula is highly activated in persons with OCD, especially in the group of patients with contamination fears [32, 36, 37].

In summary, there is strong evidence that disgust sensitivity and an over-activation in the anterior part of the insula might contribute to avoidance by increasing the aversiveness of exposure to certain stimuli, and strengthening beliefs about contamination [38]. These results suggest that a reduction of insula activity could enable a reduction in contamination anxiety in the subgroup of OCD patients who suffer predominantly from contamination fears and washing compulsions.

A Brain-Computer Interface (BCI) is a system that measures the neural activity in the central nervous system (CNS) and converts it into artificial outputs that can replace, restore, enhance, supplement, or improve a natural output [39]. BCIs can also transform brain signals into available sensory inputs that can in turn modify behavior. A variation of BCI that has recently attracted interest is the BCI-neurofeedback. With the introduction of real-time functional magnetic resonance imaging (rtfMRI) to the BCI approach (rtfMRI-neurofeedback), voluntary control over specific brain areas has been achieved both in healthy participants [40–42] and patients suffering from neuropsychiatric diseases, such as schizophrenia and depression [43–47] (for detailed reviews see [48–50]).

In a recent study, Scheinost and colleagues [51] explored the effect of rtfMRI-neurofeedback training on healthy individuals without a clinical diagnosis of anxiety disorder, but who suffered from contamination anxiety when disgust inducing stimuli were presented to them. Participants who successfully learned to control the activity of orbitofrontal cortex displayed changes in resting-state connectivity across different brain areas and an enhanced control over contamination anxiety many days after the completion the training. These results encourage the application of rtfMRI-neurofeedback in OCD patients.

Based on the above data, we designed a pilot rtfMRI-neurofeedback study in OCD patients, with two major aims. Our first aim was to investigate whether patients suffering from OCD with predominantly contamination obsessions and washing compulsions can learn to volitionally decrease (down-regulate) the BOLD activity in the anterior insula. Our second aim was to evaluate the effect of down-regulation training on clinical, behavioral and physiological changes pertaining to OCD symptoms. The further goal of this study is to ascertain the feasibility and benefits of our method for a future larger study. Hence, in addition to evaluations related to clinical symptomatology, we include three assessments designed to evaluate how self-regulation affects individual responses towards disgust inducing stimuli outside the scanner environment: 1) Ecological disgust test (EDT), conducted in real-life conditions, in which OCD patients were confronted with real, disgusting objects (e.g., chewed gums, used toilet paper), to investigate how learned down-regulation of insula modulates the patient’s ability to bear the proximity of a symptom-provoking object. 2) Disgust picture-rating test, in which patients rated disgust evoking pictures in different dimensions (valence, arousal and symptom provocation), outside the scanner, while performing down-regulation of insula without real-time feedback. In addition, physiological measures, namely, skin conductance level and heart rate variability were recorded in the same session. 3) During the rtfMRI-neurofeedback trainings, variability of heart rate and pupil size were measured in order to investigate the relationship between insula control and autonomic functions.

In view of the sample size for the pilot study, we have chosen to present individual patient data to assess the feasibility of our extensive experimental set-up, and from there to choose the most optimal clinical, behavioral, physiological, and fMRI parameters for a future larger study aimed at establishing a therapeutic intervention for OCD.

## Material and Methods

### Participants

Three adult OCD patients (2 females), diagnosed based on the Diagnostic and Statistical Manual of Mental Disorders [2] in control at the Department of Psychiatry and Psychotherapy, University of Marburg, Germany, participated in the study. Patients suffered from pronounced contamination obsessions and washing compulsions, as confirmed by a standardized clinical interview. All participants gave their written informed consent before participation. This study was approved by the ethics committee of the Faculty of Medicine, University of Marburg.

### Experimental protocol

To measure the effects of rtfMRI-neurofeedback training on OCD patients, we applied several pre- and post-training tests. Between the pre- and post-test, patients underwent several sessions (days) of rtfMRI-neurofeedback trainings. Patients were trained to down-regulate the BOLD signal extracted from the left and right anterior insula. Pre- and post-tests were based on identical experimental protocols, but in the post-test patients used the cognitive strategies that they have learned inside the scanner used to down-regulate the anterior insula. The neurofeedback training and the pre- and post-tests were conducted over a duration of 10 days for each participant (see Table 1).

**Table 1:** Experimental Protocol.

1^st^ Day	2^nd^ Day	3^rd^ Day	4^th^ -7^th^ Day	8^th^ Day	9^th^ Day	10^th^ Day
Edingburgh Handedness Scale, MWI, SCID-I, Y-BOCs, BDI-II, STAI Trait	Ecological Disgust (pre)Test	Picture ratings and physiological measurements (SCL, HR) Pre-test	rtfMRI-neurofeedback trainings and transfer runs, Inside the scanner heart rate measurements, STAI State	Ecological Disgust (post)Test	Picture ratings and physiological measurements (SCL, HR) Post-test	Y-BOCs

The neurofeedback training and the pre- and post-tests were conducted over a duration of 10 days.

### Screening

On the first day of the study, a trained clinical psychologist administered the scales and questionnaires, and obtained basic demographic information (such as gender, age, educational background), duration of the disease, previous psychotropic medications, and years using psychopharmacological medication. A vocabulary test, as a measure of verbal intelligence (Mehrfachwahl-Wortschatz-Intelligenztest, MWI) [52], and the Edinburgh Handedness Inventory [53] were also applied.

#### Pre-training clinical evaluation

The pre-training clinical evaluation included the following psychometric questionnaires and scales.

The German version of the Yale-Brown Obsessive Compulsive Scale (Y-BOCs) interview was performed to assess the severity of OCD symptoms [54]. In this test a score between 16 and 23 for compulsions and obsessions indicates moderate OCD pathology. Higher scores indicates higher levels of pathology.

The German version of the Beck Depression Inventory [55]. was used to clinically measure depression symptomatology. A score greater than 14 indicates clinically relevant depression.

The German version of the state-trait-anxiety inventory (STAI) was used for measuring trait and state anxiety [56, 57]. Higher scores are positively correlated with higher levels of anxiety.

#### Real time fMRI-BCI neurofeedback training

On the first day, before beginning the rtfMRI-neurofeedback training, a functional localizer run and a structural scan were both acquired to select the regions of interest (ROI1: left anterior insula and ROI2: right anterior insula). We used both structural and functional information, as the latter is expected to improve the accuracy of ROI selection [58], while the former is considered to be clinically more useful [59].

During the functional localizer run, 30 symptom provocative pictures and 30 neutral pictures were presented to the patients in 5 blocks. These pictures were selected from a pool which contains 150 symptom provocative (e.g., pictures of body waste, rotten food, blood injury, rats, cockroaches, dead animal corpses, vomit, etc.) and 50 neutral pictures (landscapes, objects from daily life) obtained from the International Affective Picture System (IAPS) [60] and several internet sources. Twenty–five healthy subjects rated the pictures in the dimensions of disgust, anxiety and visual complexity prior to the experiments. Pictures with the highest disgust and anxiety ratings were selected to be used in the rtfMRI-neurofeedback trainings. Selected pictures were counterbalanced according to their visual complexity. Visual complexity was defined as the level of details and the intricacy in the presented pictures. Although some of the pictures from the pool matched with other pictures in the manner of disgust and anxiety inducement levels, if their visual complexity ratings were very different; i.e., more or less complex than others, they were not selected to be included in the picture set.

Each picture lasted for 3 seconds (2 TRs). Picture presentation blocks were followed by Rest blocks in which patients were instructed to look at the plus sign on the black screen and relax. Presentation (Neurobehavioral Systems, Berkeley, CA) was used as the software for the stimulus delivery. Brain data were analyzed in real-time with the commercially available software (Turbo-BrainVoyager 3.0, Brain Innovation, Maastricht, The Netherlands) [61]. A structural T2*-weighted localizer with identical slice orientation as the functional localizer was measured with a voxel size of 2.1x2.1x2mm³. The primary motor area was selected as a reference ROI, namely ROI3, to cancel out the effect of movement related activation and global, unspecific activations, and to nullify the effect of an imprecise activation on feedback.

Each rtfMRI-neurofeedback session (day) consisted of 4 training runs. Each training run consisted of 6 alternating blocks of baseline, down-regulation and neurofeedback and reward blocks. During baseline and down-regulation blocks, the same 30 disgust inducing images in the functional localizer run were projected on a screen at the back of the MRI scanner. The projected images could be viewed by the patients via a mirror mounted on the head coil of the MRI.

**Fig 1:**
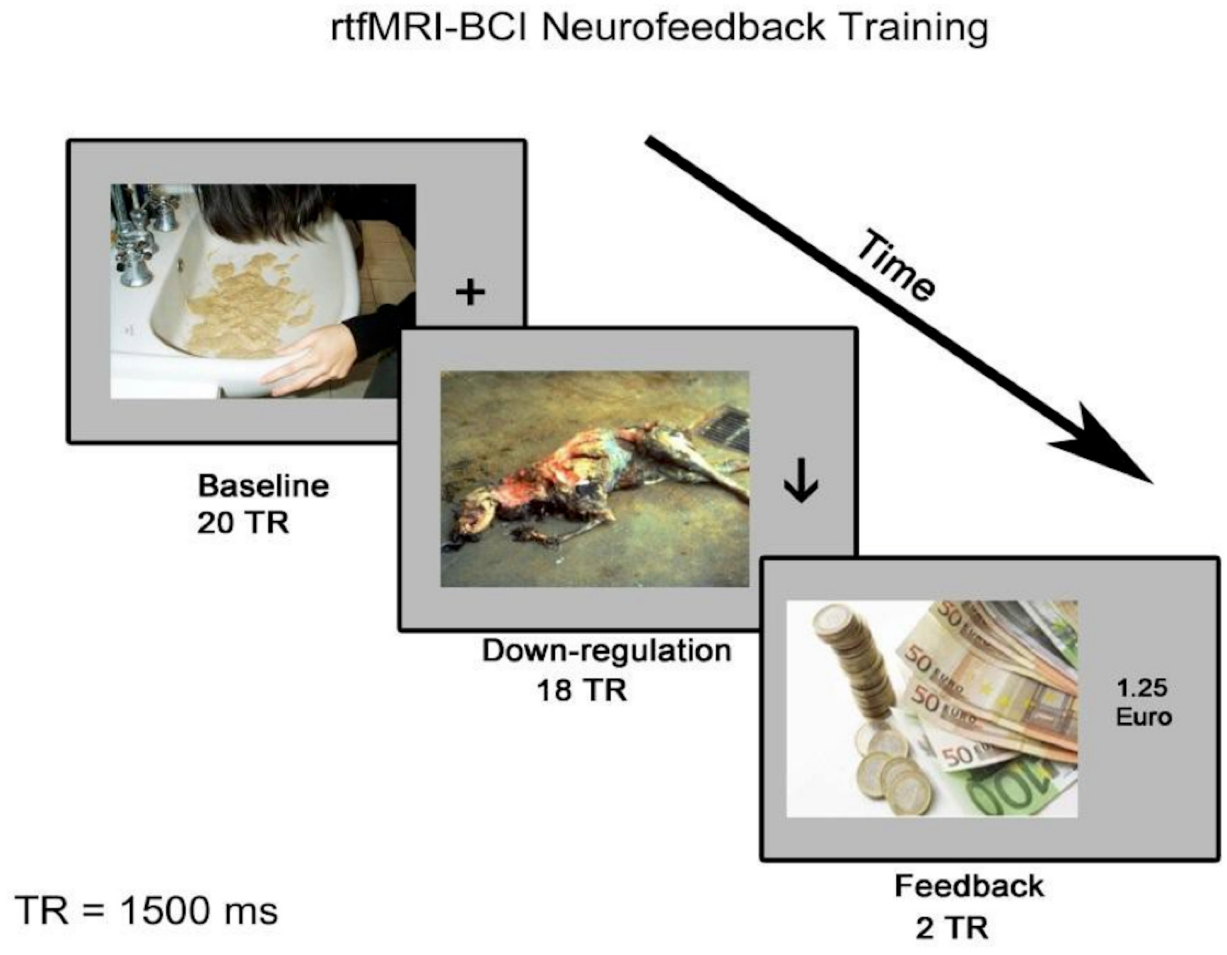
Flow of the rtfMRI-neurofeedback training run. Each run consisted of 6 baseline (duration: 30 seconds each) and 6 down-regulation blocks (duration: 27 seconds each). Immediately following each block of down-regulation, a monetary feedback was presented for 3 seconds. The same design was used for the transfer runs (5^th^ run of the each session) but no feedback was presented.

Baseline blocks were indicated by the discriminating stimulus, plus sign (‘+’), on the right side of each disgust inducing image. Every block lasted for 30 seconds during which participants were instructed to look directly at the screen, view the images, and not suppress the feelings triggered by these images. For the down-regulation blocks (indicated by the discriminating stimulus, down arrow (↓), on the right side of the picture, 27 seconds each), participants were instructed to find an effective cognitive strategy to decrease the feedback signal while viewing the images. No specific cognitive strategy was suggested to the participants. Every image was used for both baseline and down-regulation blocks separately at least once through the runs. Fig 1 shows the flow chart of the rtfMRI-neurofeedback training runs.

The neurofeedback protocol was based on operant conditioning, wherein the visual display of monetary reward at the end of each neurofeedback trial served as the reinforcement. Reinforcement was calculated and presented immediately following each down-regulation block (at the interval of 3 seconds). The amount of monetary feedback was calculated by using the following equation:

$$f=C\;\left\{\text{mean}{\left(\frac{ROI_{1}+ROI_{2}}{2}-ROI_{3}\right)}_{baseline}-\text{mean}{\left(\frac{ROI_{1}+ROI_{2}}{2}-ROI_{3}\right)}_{regulation}\right\}$$

where, C was an arbitrary but fixed proportionality constant. A positive feedback value was presented when the mean BOLD value was reduced during the down-regulation condition relative to the baseline condition. If the mean BOLD level during down-regulation was the same or higher than the baseline value, the resulting feedback was presented as zero. Due to the potentially disturbing effect of the disgust pictures presented inside the scanner and the anxiety which might be triggered by the scanning process itself, we did not want to further induce a negative emotion led by either negative reinforcement or punishment. Hence, we presented only positive feedback values which signals to the participants that their response is correct [62].

Training runs with contingent feedback were interspersed with “transfer runs” in which the patients were asked to down-regulate the ROIs without the aid of the feedback, and by using the mental strategy learned during the neurofeedback training. Transfer runs were included in order to train subjects to self-regulate in a real-world environment (outside the scanner). Twelve pictures from the neurofeedback runs were used as stimuli in the transfer run.

Brain data were analyzed in real-time with the Turbo-BrainVoyager [61], which includes on-line incremental 3D motion detection and correction, and drift removal. The software is capable of incrementally computing statistical maps based on the General Linear Model (GLM) and event-related averages. The visual feedback (“monetary reward”) was calculated from brain activity using Matlab 2012b (The MathWorks, Natick, MA) software running on a separate computer connected via a local area network (LAN) to the scanner and to the Turbo-Brain Voyager computer [63].

During the rtfMRI-neurofeedback training sessions heart rate (HR) measures were collected as an indicator of the OCD related sympathetic-parasympathetic activity. Heart rate variability (HRV) was monitored using a pulse oximeter. Inter-beat intervals were converted to heart rate in beats per minute (BPM). The distributions of the data were checked for normality with Shapiro-Wilk test. Differences between the baseline and the down-regulation conditions in the pre-test were statistically compared with the differences between the baseline and the down-regulation conditions in the post-test by applying paired samples t-tests with SPSS v19 (IBM, Armonk, NY).

Patients completed the German version of State-Trait Anxiety Inventory (STAI-State) before and after each session as a measure of their anxiety levels related to the fMRI measurement.

#### MR acquisition

MRI scans were acquired using a 3.0 Tesla body scanner, with standard 12-channel head coil (Siemens Magnetom Tim TRIO, Siemens, Erlangen, Germany) at the Department of Psychiatry, University of Marburg, Germany. A standard echo-planar imaging sequence was used for acquiring functional images (EPI; TR: 1.5 s, TE: 30 ms, matrix size: 64x64, flip angle α: 90°). Each volume consisted of 16 axial slices (voxel size: 3.3 x 3.3 x 5.0 mm^3^, slice gap of 1 mm) in AC-PC alignment. For the structural localizer we used T2*w images (56 axial slices, voxel size 2.1 x 2.1 x 2mm³, gap of 0.4 mm, TR: 4.5 s, TE: 35ms, matrix size: 100x100, flip angle α: 90°). In order to superimpose functional maps on brain anatomy, a high-resolution T1-weighted structural scan was collected in each session (MPRAGE, matrix size: 256 x 256, 176 sagittal slices, 1mm³ isotropic voxels, TR = 1900 ms, TE: 2.52 ms, TI: 900 ms, flip angle α: 9°).

#### Offline fMRI analysis

Hypothesis driven ROI analysis was performed using the ROIs previously selected for each patient during the rtfMRI sessions/neurofeedback runs. We performed fMRI pre-processing and statistical analysis using the FMRIB Software Library (FSL, http://www.fmrib.ox.ac.uk/fsl, Centre for Functional MRI of the Brain, Oxford University, UK). Data processing included rejection of the first 10 volumes, motion correction (MCFLIRT), slice timing correction, spatial smoothing (full width half maximum, 5mm) and high pass filtering (cutoff frequency 1/60Hz). Z (Gaussianised T/F) statistic images were thresholded using clusters determined by Z >2.3 and and Z <-2.3 and a (corrected) cluster significance threshold of P = 0.05.

FMRI data analysis packages like FSL, based on the GLM method, are more sensitive to mean differences in the BOLD signals between experimental conditions, and hence are more suitable for finding statistically significant differences in brain activity when neurofeedback training is sufficiently long and effective. However, the GLM is less sensitive to trial-by-trial variations in the form of systematic increases or decreases during the course of neurofeedback training. Furthermore, earlier studies by our group [41] showed that down-regulation of insula is relatively more difficult to achieve than up-regulation, especially when shorter durations of neurofeedback training was employed. In consideration of the above, we wanted to develop a more sensitive measure of down-regulation that can potentially detect a learning effect due to neurofeedback training. Towards this end, we compared the BOLD signal level in the ROIs for every repetition time (TR) during the down-regulation blocks with the mean BOLD level during the previous baseline block. If the BOLD signal level in the down-regulation block was lower than the mean BOLD of the previous baseline block for that particular TR, it was counted as a "hit". Because each down-regulation block lasted 27 seconds (18 TRs), and there were 6 blocks through one run, the maximum number of hits for one run could be 108. We counted every hit of every down-regulation block during the run, and converted the total number of hits into the percentages. We named this analysis as the “hits analysis”.

We reported results of both the GLM and the percentage of hits analyses for each patient, separately.

During the rtfMRI-neurofeedback training sessions, eye-tracker recordings (SR-Research, Eyelink 1000) were performed to evaluate whether patients were attending to the presented pictures during baseline and down-regulation conditions. Recordings indicated that patients attended to the pictures during the training sessions. None of the patients kept their eyes closed during any part of the experiment.

#### Pre and Post behavioral tests

These tests were conducted in each participant before and after the rtfMRI-neurofeedback training. Statistical analysis of behavioral and physiological data was performed by applying repeated measures two-way ANOVA (factors: time and condition) and post-hoc t-tests.

#### 1. Ecological disgust test (EDT)

The EDT was designed to evaluate each patient’s response to a symptom-provoking stimulus in a naturalistic environment. Outside the scanner and in a separate room, patients were shown real-world disgusting objects, selected individually for each patient according to his/her own self-reports during the screening session. The object was placed in the first position on a wheeled table at a distance of 5 meters apart from the patient. On the left side of this object, a clearly visible ‘+’ sign and or a ‘↓’ sign sign was shown. In each trial, the selected object was slowly brought towards the patient at a constant pace, either with the ‘+’ sign or with the ‘↓’ sign. There were 20 trials of this kind, each sign being presented 10 times. Patients were instructed to focus on the object, and say ‘stop’ when they feel that the object should not come any closer. As long as the patient does not say ‘stop’, the experimenter would move closer, and at the end bring the object in contact with the patient’s hand. The distances between the starting point and the point at the “stop” moment were measured for ‘+’ signed and ‘↓’ signed blocks along the direction of movement of the experimenter for both pre-test and post-tests. The meanings of the signs (‘+’ = baseline, ‘↓’ = insula down regulation) were not explained to the patients in the pre-test sessions. In the post-test, the same procedure and objects were used, but this time participants were instructed to use the cognitive strategies that they had learned during rtfMRI-neurofeedback trainings. Thus, in the above tests, participants were instructed to recreate mental strategies that they had used during the neurofeedback training of down-regulation (during presentation of the discriminating stimulus ‘↓’) of anterior insula, but now in the absence of feedback and in real-life conditions.

We expected that down-regulation of anterior insula would allow the patient to stay closer to the disgust object than during baseline condition (once down-regulation was learned, i.e., in the post-test).

**Fig 2:**
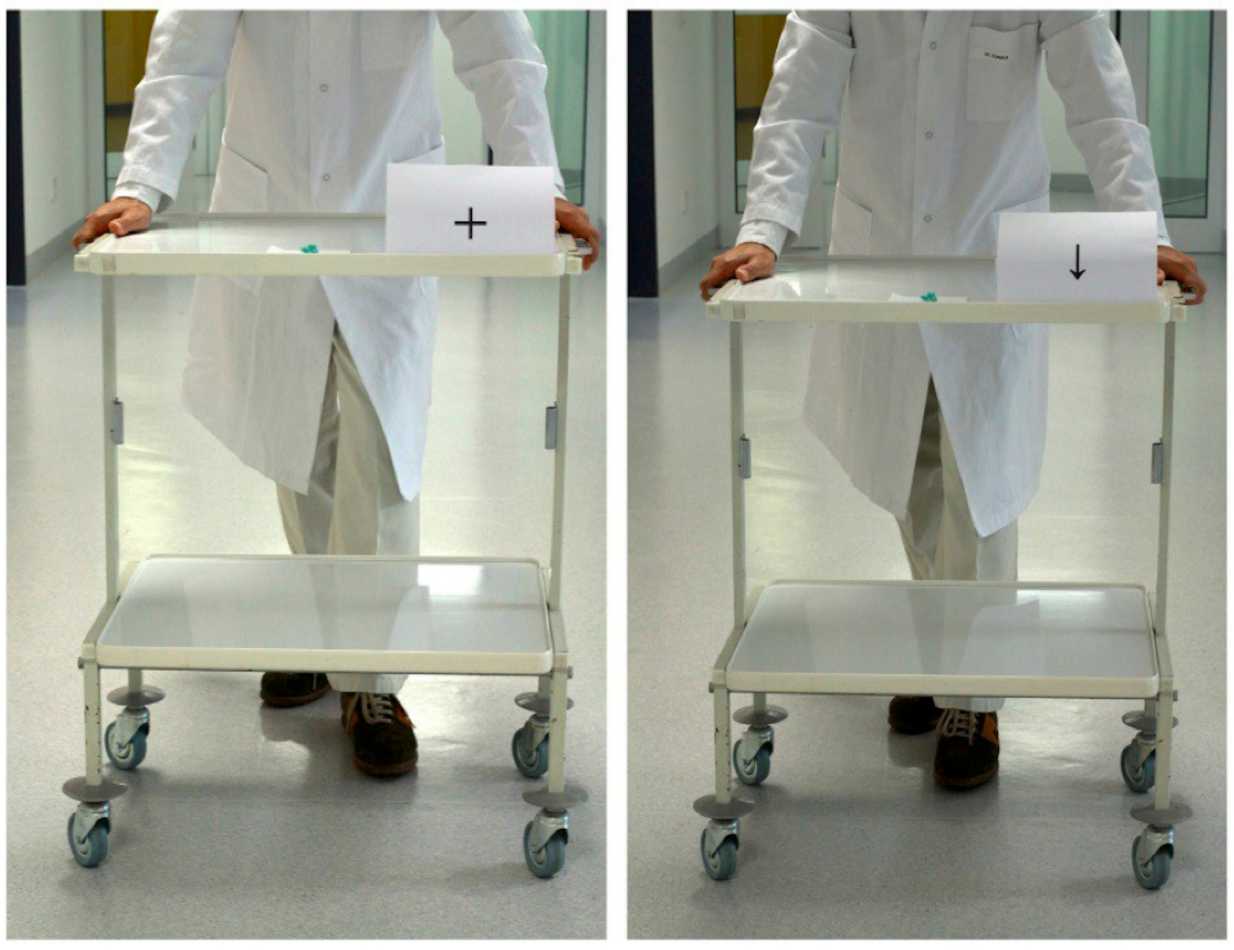
The Ecological Disgust Test and the flow of the experiment. The experimenter approached the patient with a real-life, disgust-inducing object from a distance of 5 meters either with a ‘↓’ or ‘+’ cue (10 times each). The patient focused on the object (two newly chewed gums in this picture) as the experimenter approached him/her, and said “stop” whenever he/she felt that the object should not come any closer. In the pre-test, the meanings of the cues are not explained to the patients. In the post-test, during down-arrowed runs, patients used the cognitive strategies that they had learned in the rtfMRI-neurofeedback training sessions.

Fig 2 shows the materials used in the Ecological Disgust Test.

#### 2. Picture rating test

We tested patient responses to disgusting pictures in a naturalistic environment, outside the scanner, using a set of visual contamination, anxiety inducing stimuli. During the pre-test, another 50 pictures (30 contamination anxiety related, 20 neutral) taken from our previlously constructed picture pool were presented to the patients (15 seconds each) two times, in two separate runs, in a counterbalanced order via a 21.5" LCD monitor. Patients were shown either a down arrow (‘↓’) or a plus sign (‘+’) on the right side of the screen attached to each picture. Pictures that were presented with the (‘+’) sign (baseline condition) in the first run were presented with the (‘↓’) sign (down-regulation condition) in the second run. The meanings of these signs were not explained to the patients in the pre-test. Patients were asked to rate the pictures after each presentation in three dimensions, i.e. valence, arousal and OCD-symptom provocation by using a visual analogue scale (VAS) [64]. with the help of the computer mouse. The positions of respondents’ marks on the VAS were scaled as distinct points, resulting in codes from 1 to 1900. In the post-test, the same procedure was used, except for the fact that during the down-regulation conditions, patients were instructed to use the cognitive strategies that they had learned in rtfMRI-neurofeedback training.

We expected a modulation of the ratings toward the pictures during down-regulation compared to baseline (once down-regulation was learned, i.e., in the post-test).

**Fig 3:**
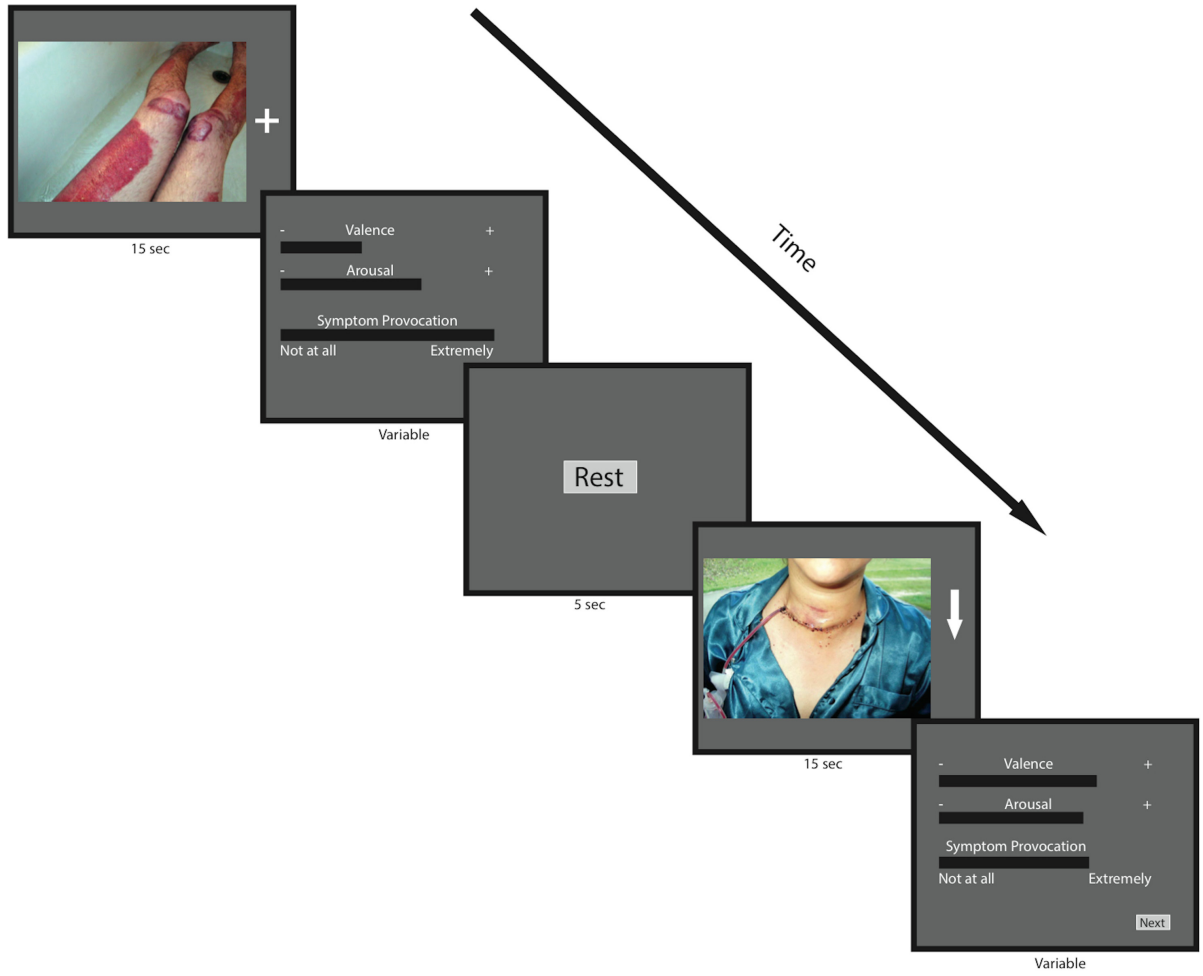
Picture-rating test. Patients rate each picture at the dimensions of valence, arousal and OCD symptom provocation. During the ratings, skin conductance level and heart rate data were collected.

Fig 3 shows the Picture Rating Test and the flow of the experiment.

#### 3. Physiological measures during picture ratings (SCL & HR)

Physiological responses, i.e, skin conductance level (SCL) and heart rate (HR) were measured by the NeXus-32 Wireless Physiological Monitoring and Feedback System (Mind Media BV, The Netherlands) during the picture-rating test. Skin conductance was measured from 2 electrodes attached to the palms of the non-dominant hand. Mean skin conductance levels were calculated for two conditions (disgusting picture-baseline, disgusting picture-down-regulation, 15 seconds each). Heart rate variability was monitored using a pulse oximeter. Inter beat intervals were converted to heart rate in beats per minute.

On the 8^th^, 9^th^ and 10^th^ days of the study (6^th^, 7^th^ and the 8^th^ days for the first two patients) the post-tests (EDT, picture rating, SCL/HR measurements) were performed.

After the post-tests, the assessment of the general psychopathology was performed again (as in the pre-training clinical evaluation).

To differentiate the effects of variables other than neurofeedback training on the behavioral and physiological results e.g., habituation to the pictures/disgust stimuli, medication or other therapeutics, we compared the differences between baseline and down-regulation conditions (interspersed in the same runs) for both pre-test and post-test. Because the patients did not know the meanings of the (‘↓’) or (‘+’) signs in the pre-test, we did not expect any difference between baseline and down-regulation conditions in the pre-test.

## Results

In this section, we shall elaborate on the results of each patient’s pre-test, rtfMRI-neu**r**ofeedback training and post-test, separately. We have chosen to present individual patient data instead of group data due to the small sample size of this pilot study.

Patients No. 1 and No. 2 participated for 2 days in the rtfMRI-neurofeedback training (on the 4^th^ and 5^th^ days of their measurements) and continued with the post-tests on the 6^th^, 7^th^ and 8^th^ days, while patient 3 participated in four days of neurofeedback training and then continued with the post-tests on 8^th^, 9^th^ and 10^th^ days.

### Patient 1

Patient 1 was a 19-year-old right-handed female. She was undergoing cognitive behavioral psychotherapy for 3 weeks during the measurements, and was not using psychotropic medication.

According to SCID-I interview, Patient 1 met the criteria for OCD with predominantly compulsive behavior. The Y-BOCs score also indicated mild OCD symptoms at the pre-test and the post-test. The BDI-II indicated severe depression. In comparison to healthy subjects, the patient suffered from higher anxiety levels according to the STAI trait.

Patient’s pre-post questionnaire results are presented in Table 2.

**Table 2:** Clinical evaluation of the Patient 1.

TEST	SCID I	Y-BOCs	BDI-II	STAI Trait	STAI State
**Pre-test**	F42.1 OCD, Predominantly compulsive acts	12	42	76	Pre-scan 69**,** Post-scan 68
**Post-test**	-	11	-	-	Pre-scan 48, Post-scan 40

### Real-time fMRI-BCI neurofeedback training analysis

#### Offline fMRI analysis

Due to technical difficulties, Patient 1 could complete only two runs of rtfMRI-neurofeedback training on the first day of rtfMRI-neurofeedback training.

Comparison of brain activity of Patient 1 for the down-regulation condition between the 1^st^ and 2^nd^ days showed increased activity in right superior frontal gyrus, right middle temporal gyrus, left postcentral gyrus, medial frontal cortex, brain stem and middle temporal gyrus/temporooccipital part on the second day of rtfMRI-neurofeedback training. Activity decreases on the second day as compared with the first day were observed in the supplementary motor area (SMA), precentral gyrus, anterior and posterior cingulate gyrus, middle frontal gyrus, left posterior supramarginal gyrus, orbitofrontal cortex (OFC), anterior insula, superior temporal gyrus, frontal pole, left and right caudate, left precuneus, lingual gyrus and occipital pole.

**Fig 4:**
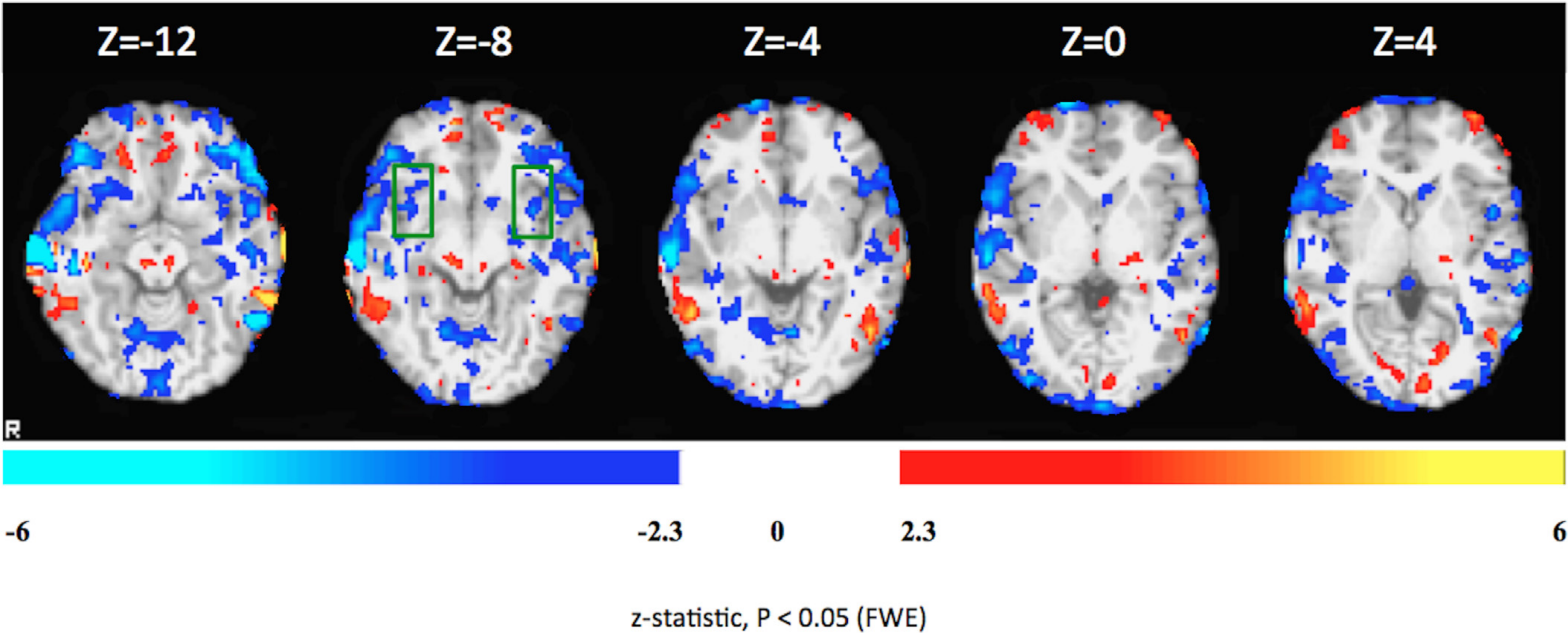
Differential brain activations during the down-regulation condition in Patient 1 on the first and second days of rtfMRI-neurofeedback training. Colors red-yellow: increased activity on the second day as compared to the first day. Blue-white: decreased activation the second day as compared to the first day. The colored functional maps were overlaid on T1-weighted structural images of four representative axial brain sections covering insula, which is delineated by the green rectangle. Statistical significance was based on z-statistic threshold of -2.3 and 2.3 followed by multiple comparisons correction at the cluster level using Family-Wise Error (FWE) at p < 0.05.

Fig 4 shows comparison of brain activity for the down-regulation condition between the 1^st^ and 2^nd^ days of the rtfMRI-neurofeedback training.

#### Hits analysis

**Fig 5:**
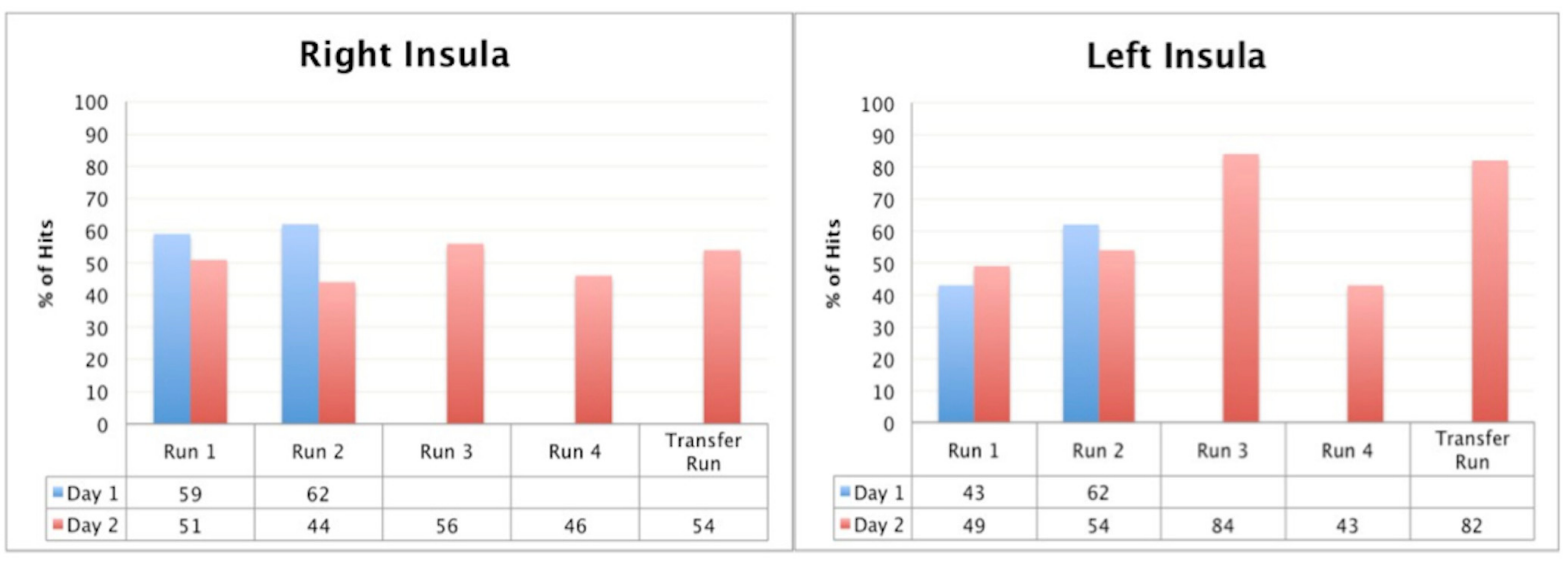
Patient 1, the percentage of the hits from the right and the left anterior insula through rtfMRI-neurofeedback trainings respectively. Blue columns show the percentage of the hits of the first, red columns show the second session of rtfMRI-neurofeedback training run by run. Due to technical difficulties, the first patient could complete only two runs of rtfMRI-neurofeedback training on the first session of rtfMRI-neurofeedback training.

Fig 5 shows the percentage of hits for Patient 1 achieved during rtfMRI-neurofeedback training and the transfer runs from right and left anterior insula, respectively. On the second day, in the transfer run, the patient had 89 hits in the left insula, which corresponds to 82% of the possible total hits in a run. The patient used “thinking funny stories” as her cognitive strategy for down-regulation.

Statistical comparisons (Wilcoxon signed-rank test) showed no significant diffrence between the 1^st^ and 2^nd^ day of trainings both for the right insula (day 1 mean number of hits = 60.5, sd = 2.12; day 2 mean number of hits = 50.2, sd = 5.11; p = 0.18), and the left insula (day 1 mean number of hits = 52.5, sd = 13.45; day 2 left mean number of hits = 62.4, sd = 19.2; p = 0.655).

No statistically significant difference was found between the left and right insula for both 1^st^ (p = 0.317) and the 2^nd^ day (p = 0.223) as well.

#### Heart rate measurements in the scanner

The first patient’s HR scores as beats per minute (BPM) for baseline (M = 90.03, sd = 1.67), and down-regulation (M = 87.25, sd = 1.68) conditions on the first day of training were significantly different, t(11) = 5.528, p<0.01. There was also a significant difference between baseline (M = 91.24, sd = 3.79) and down-regulation (M = 90.36, sd = 3.87) conditions in the second day of training, t(23) = 2.275, p<0.05. In both days, HR was lower during the down-regulation blocks compared to the baseline blocks.

#### Ecological disgust test

In the EDT, two newly chewed gums were used as real-life, disgust objects. The distance between the starting point and the point at the “stop” moment were measured as meters for ‘+’ signed baseline and ‘↓’ signed down-regulation blocks for both pre-test and post-tests. We had 5 baseline and 5 down-regulation blocks in the pre-test for this patient.

Repeated mesures two-way ANOVA analysis showed significant effect of time F(1, 4) = 25.62, p<0.01 and condition F(1, 4) = 14.18, p = 0.02. The interaction between the time and the condition was significat as well F(1, 4) = 7.323, p = 0.05.

Post-hoc paired sample t-tests of the pre-test showed that there was no significant difference between baseline and down-regulation conditions in the patient’s response to the disgust objects. A significant difference between baseline (M = 4.45 meters, sd = 0.32) and down-regulation (M = 4.89 meters, sd = 0.27) conditions was found in the post-test, t(9) = -9.258, p<0.01. During the down-regulation conditions, the experimenter moved closer to the patient with the disgust object. On 3 post-test down-regulation trials, the patient allowed the experimenter to touch her hand with the chewed gum. The patient did not allow the experimenter to touch her with the chewed gum during the baseline trials.

#### Picture ratings

Repeated mesures two-way ANOVA analysis results showed significant effect of time F(1, 31) = 14.31, p<0.01 on the arousal ratings. No significant effect of condition F(1, 31) = 0.224, p = 0.639 and no interaction between time and condition was observed F(1, 31) = 0.133, p = 0.718.

We found significant effect of time F(1, 31) = 89.85, p<0.01 and the condition F(1, 31) = 23.04, p<0.01 on the valence ratings. Also, the interaction between time and the condition was found significant F(1, 31) = 45.62, p<0.01.

Significant effect of time F(1, 31) = 119.18, p<0.01 and condition F(1, 31) = 40.75, p<0.01 on the symptom provocation ratings were also observed. There was a significant interaction between the time and the condition for the symptom provocation ratings F(1, 31) = 81.24, p<0.01.

In the post-test, the patient rated the pictures as having less negative valence and as being less symptom provocative following the down-regulation blocks (p<0.01 for both conditions).

#### Physiological measures during picture ratings (SCL & HR)

No statistically significant difference was observed in SCL between baseline (M = 12.22, sd = 0.59) and down-regulation conditions (M = 12.13, sd = 0.43) in the pre-test in the picture rating test. The differences between baseline (M = 17.26, sd = 7.61) and down-regulation (M = 15.03, sd = 3.71) conditions did not reach significance in the post-test either. HR score diffrences as BPM between baseline (M = 88.49, sd = 2.13) and down-regulation (M = 86.82, sd = 2.48) conditions in the pre-test were not significant. Also, no significant difference was observed between baseline (M = 80.10, sd = 3.16) and down-regulation (M = 79.79, sd = 1.61) conditions in the post-test.

### Patient 2

Patient 2 was a 25 years old right handed female. She was an in-patient, underwent cognitive behavioral psychotherapy for 3 weeks before the measurements and was using psychotropic medication (Setralin 150 mg).

Patient 2 met SCID-I criteria for OCD predominantly with compulsive behavior, and had a low severity of symtomatology according to Y-BOCs. No clinically significant depression or anxiety was detected.

The patient’s pre-post clinical results are presented in Table 3.

**Table 3:** Clinical evaluation of the Patient 2.

TEST	SCID I	Y-BOCs	BDI-II	STAI Trait	STAI State
**Pre-test**	F42.1 OCD, Predominantly compulsive acts.	6	0	51	Pre-scan 40**,** Post-scan 40
**Post-test**	-	9	-	-	Pre-scan 31**,** Post-scan 42

### Real-time fMRI-BCI neurofeedback training analysis

#### Offline fMRI analysis

Between the first and the second day of rtfMRI-neurofeedback trainings, we observed an increase in activity in the left postcentral gyrus, posterior cingulate gyrus, right hippocampus and subcallosal cortex on the second day. Deactivations were observed on the second day as compared with the first day in SMA, anterior cingulate gyrus, middle frontal gyrus, frontal pole, thalamus, caudate, inferior frontal gyrus, OFC, insula, putamen, middle temporal gyrus, occipital fusiform gyrus, occipital pole and the lingual gyrus.

**Fig 6:**
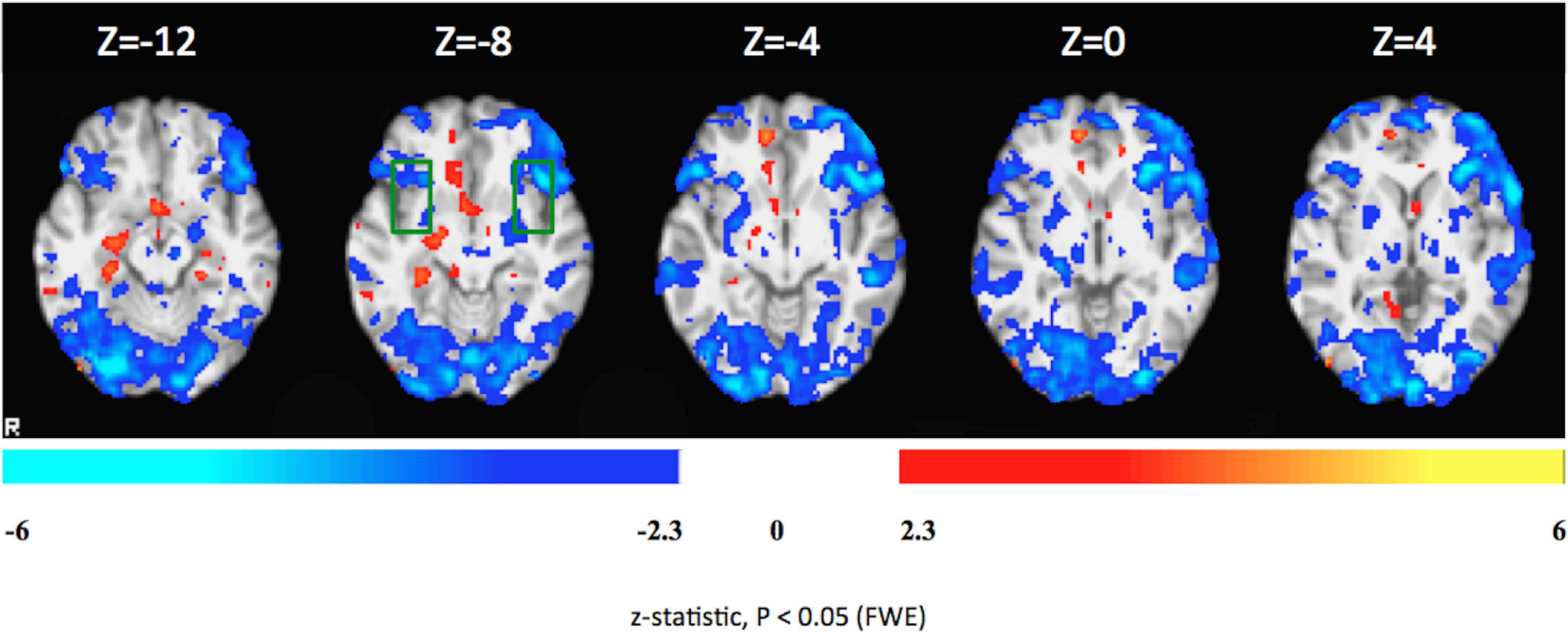
Differential brain activations during the down-regulation condition in Patient 2 on the first and second days of rtfMRI-neurofeedback training. Colors red-yellow: increased activity on the second day as compared to the first day. Blue-white: decreased activation the second day as compared to the first day. The colored functional maps were overlaid on T1-weighted structural images of four representative axial brain sections covering insula, which is delineated by the green rectangle. Statistical significance was based on z-statistic threshold of -2.3 and 2.3 followed by multiple comparisons correction at the cluster level using Family-Wise Error (FWE) at p < 0.05.

Fig 6 shows comparison of brain activity for the down-regulation condition between the 1^st^ and 2^nd^ days of the rtfMRI-neurofeedback training.

#### Hits analysis

The patient showed similar down-regulation performances on the first and the second days of the rtfMRI-neurofeedback trainings. On the first day, during the transfer runs, she achieved 65 hits (60%) and 71 hits (65%) in the down-regulation of the right and the left insula, respectively. On the second day, the number of hits from the right insula decreased to 56 (51%) but an increase in the number of hits 75 (69%) was observed for left insula.

Statistical comparisons showed no significant difference between the 1^st^ and 2^nd^ day of trainings both for the right insula (day 1 mean number of hits = 71, sd = 14.3; day 2 mean number of hits = 69.6, sd = 14.9; p = 0.686), and the left insula (day 1 mean number of hits = 70, sd = 13.3; day 2 mean number of hits = 71.8, sd = 16.8, p = 0.785).

No statistically significant difference was found between the left and right insula for both the 1^st^ (p = 0.465) and the 2^nd^ days (p = 0.786).

The patient reported “thinking herself lying on the grass” as her cognitive strategy for down-regulation conditions.

**Fig 7:**
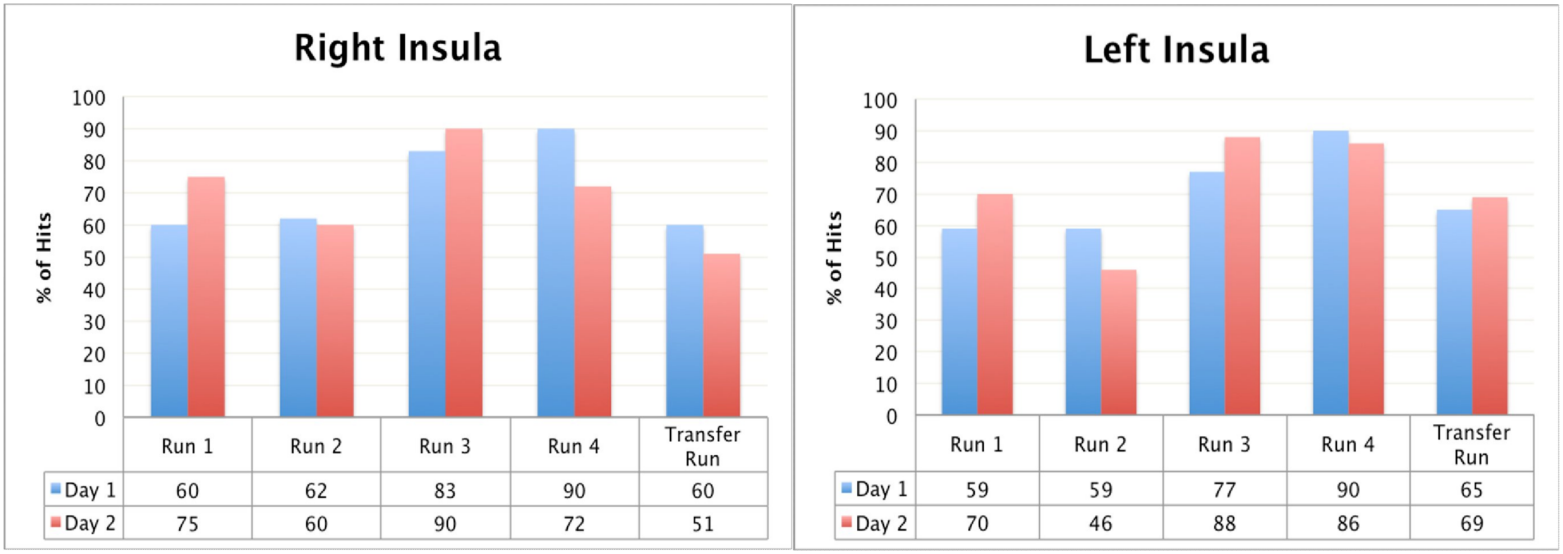
Patient 2, the percentage of the hits from the right and the left anterior insula through rtfMRI-neurofeedback trainings respectively. Blue columns show the percentage of the hits of the first, red columns show the second session of rtfMRI-neurofeedback training run by run.

Fig 7 shows the percentage of the hits that the patient had through rtfMRI-neurofeedback training and the transfer runs from right anterior insula and left anterior insula, respectively.

#### Heart rate measurements in the scanner

Patient’s HR scores as BPM for baseline (M = 82.20, sd = 1.46) and down-regulation (M = 80.97, sd = 2.25) conditions on the first day of training were significantly different, t(23) = 2.692, p<0.05. In the down-regulation condition, the patient showed significantly lower HR compared to the baseline condition. On the second day of training, although the patient continued to show a lower HR in the down-regulation condition, the difference between the baseline (M = 93.76, sd = 2.79) and the down-regulation (M = 92.92, sd = 2.66) conditions did not reach significance.

#### Ecological Disgust Test

In the EDT trials, used toilet papers, taken from a toilet bin and placed inside an open-mouthed nylon bag, were used as disgust objects.

Repeated mesures two-way ANOVA analysis showed significant effect of time F(1, 9) = 45.92, p<0.01. However no significant effect of condition F(1, 9) = 2.273, p = 0.166 and time x condition interaction F(1, 9) = 0.115, p = 0.743 was observed.

#### Picture rating test

Repeated mesures two-way ANOVA analysis showed significant effect of time F(1, 29) = 16.91, p<0.01 on the arousal ratings. No effect of condition F(1, 29) = 0.01, p = 0.92 and interaction between time and condition was observed F(1, 29) = 1.41, p = 0.244.

Significant effect of time F(1, 29) = 20.89, p<0.01 was found on the valence ratings as well. However, no effect of condition F(1, 29) = 0.218, p = 0.644 and no significant interaction between the time and the condition F(1, 29) = 0.006, p = 0.940 was observed.

Effects of time on the symptom provocation ratings was significant F(1, 29) = 14.338, p<0.01. No significant effect of condition F(1, 29) = 0.076, p = 0.785 and no interaction effect between the time and the condition was found (1, 29) = 0.288, p = 0.596.

#### Physiological measures during picture ratings (SCL & HR)

The difference in SCR between the baseline (M = 4.83, sd = 0.9) and the down-regulation (M = 5.21, sd = 0.98) conditions was significant in the pre-test (p = 0.013). SCL was higher during down-regulation conditions than the baseline condition. In the post-test SCL differences between the baseline (M = 2.66, sd = 0.51) and the down-regulation (M = 2.56, sd = 0.63) conditions were not significantly different.

HR difference as BPM between baseline (M = 81.89, sd = 4.24) and the down-regulation (M = 81.84, sd = 3.60) in the pre-test was not significant, but we found a significant difference between the conditions in the post-test (p = 0.013). HR was higher in the down-regulation (M = 90.73, sd = 3.25) condition as compared to the baseline (M = 89.36, sd = 2.43).

### Patient 3

The third patient was a 26-year old left handed male. He was under psychotropic medication (Setralin 125 mg., Risperdal 0.5 mg.), but not undergoing psychotherapy during the measurements. This patient received 4 days of rtfMRI-neurofeedback training.

Patient 3 met criteria for OCD and major depression in accordance with BDI-II. A decrease in the Y-BOCs score was observed after training. Patient 3 showed high levels of anxiety before and during the experiments. Patient’s pre-post questionnaire results are presented in Table 4.

**Table 4:** Clinical evaluation of the Patient 3.

SCID I	Y-BOCS	BDI-II	STAI Trait	STAI State
F42.2 OCD, Mixed obsessional thoughts and compulsive acts. Major Depression	Pre-test 36, Post-test 33	35	79	Pre-scan 57**,** Post-scan 57,Pre-scan 54**,** Post-scan 55, Pre-scan 65**,** Post-scan 55, Pre-scan 60**,** Post-scan 53

### Real-time fMRI-BCI neurofeedback training analysis

#### Offline fMRI analysis

As compared with the 1^st^ day, Patient 3 showed significantly increased BOLD responses during the down-regulation condition in the left lateral occipital cortex/superior division left, hippocampus, right amygdala and brain stem on the 4^th^ day.

Statistical comparisons of the 1^st^ and the 4^th^ days yielded decreased activity in superior frontal gyrus, SMA, middle frontal gyrus, precentral gyrus, posterior supramarginal gyrus, anterior and posterior cingulate gyrus, precuneus, insula, putamen, caudate, thalamus, OFC, frontal pole, occipital pole, posterior middle temporal gyrus, right anterior supramarginal gyrus and precental gyrus.

**Fig 8:**
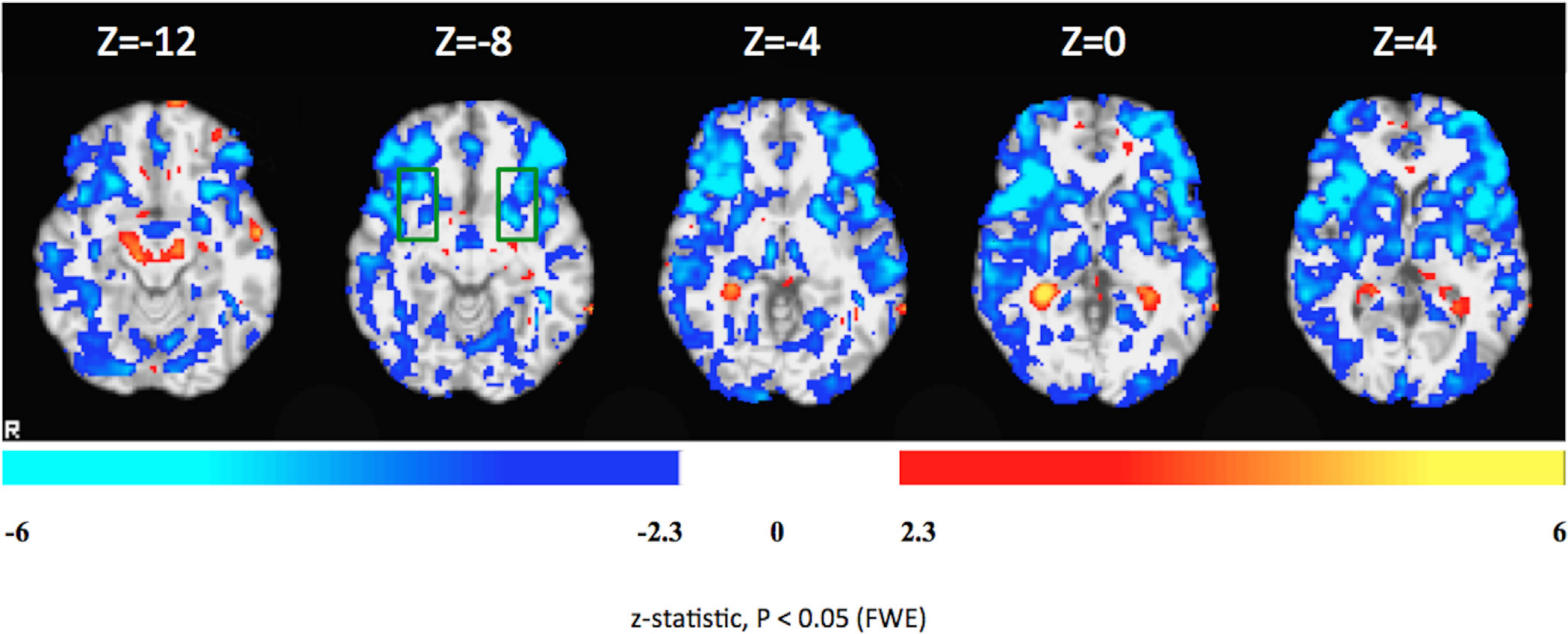
Differential brain activations during the down-regulation condition in Patient 3 on the first and second days of rtfMRI-neurofeedback training. Colors red-yellow: increased activity on the second day as compared to the first day. Blue-white: decreased activation the second day as compared to the first day. The colored functional maps were overlaid on T1-weighted structural images of four representative axial brain sections covering insula, which is delineated by the green rectangle. Statistical significance was based on z-statistic threshold of -2.3 and 2.3 followed by multiple comparisons correction at the cluster level using Family-Wise Error (FWE) at p < 0.05.

Fig 8 shows comparison between the 1^st^ and 4^th^ days of FSL analyses of the rtfMRI-neurofeedback training.

#### Hits analysis

Statistical comparisons showed no significant diffrence between the 1^st^ and 4^th^ day of trainings both for the right insula (day 1 mean number of hits = 65.8, sd = 10.9; day 4 mean number of hits = 69.8, sd = 15.1; p = 0.354), and the left insula (day 1 mean number of hits = 51.8, sd = 18.8; day 4 mean number of hits = 54.8, sd = 17.3; p = 0.686).

No statistically significant difference was found between the left and the right insula for both 1^st^ (p = 0.225) and 4^th^ day (p = 0.43).

As the mental strategy for down-regulation, the patient mentally replaced the “ugly” parts in the pictures with “nicer” objects. On the third day of training the patient changed his strategy through the runs (e.g., thinking on nice memories and concentrating on colors of the objects). In this session, his overall number of hits was the lowest as compared with the other days.

**Fig 9:**
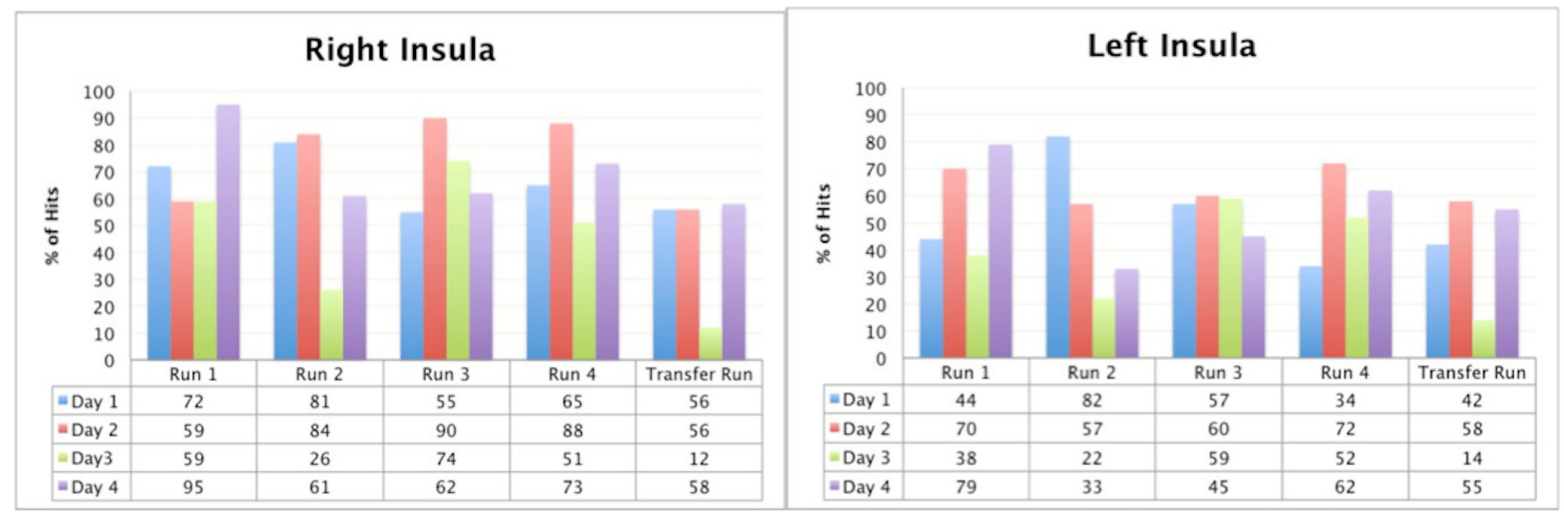
Patient 3, the percentage of the hits from the right and the left anterior insula through rtfMRI- neurofeedback trainings respectively. Blue columns show the percentage of the hits of the first, red columns show the second, green columns show the third and purple columns show the fourth session of rtfMRI-neurofeedback training run by run.

Fig 9 shows the percentage of the hits that patient had through rtfMRI-neurofeedback training and the transfer runs for four days.

#### Heart rate measurements in the scanner

In contrast to the previous two patients, the third patient showed increased HR during the down-regulation conditions as compared to the baseline through the rtfMRI-neurofeedback training sessions. On the first day of the training, the scores as BPM for baseline (M = 72.89, sd = 1.26) and down-regulation (M = 73.89, sd = 1.64) conditions were significantly different, t(17) = -2.359, p<0.05. There was also a significant difference between baseline (M = 78.51, sd = 1.87) and down-regulation (M = 79.21, sd = 2.19) conditions on the second day of trainings, t(23) = -2.536, p<0.05. For the third day, no significant difference was found between baseline (M = 79.43, sd = 1.98) and regulation (M = 80.21, sd = 2.24) conditions. Likewise, there was no significant difference between baseline (M = 83.45, sd = 3.25) and down-regulation (M = 83.91, sd = 3.69) conditions on the fourth day.

#### Ecological Disgust Test

For this patient, used toilet papers were the real-life disgust objects (similar to that of the second patient). These disgust objects were put into a nylon bag and the nylon bag’s mouth was left open.

Repeated mesures two-way ANOVA analysis results showed significant time F(1, 9) = 10.028, p = 0.01 condition F(1, 9) = 37.166, p<0.01 and time x condition interaction F(1, 9) = 11.423, p<0.01 effects on EDT.

Post-hoc paired samples t-tests revealed that, the difference in the patient’s response between baseline (M = 4.25 meters, sd = 0.10) and down-regulation (M = 4.27meters, sd = 0.06) conditions in pre-test did not reach significance. There was a significant difference in the patient’s response between baseline (M = 4.26 meters, sd = 0.13) and down-regulation (M = 4.49 meters, sd = 0.15) conditions in the post-test, t(9) = -5.314, p<0.01. The experimenter could get closer to the patient with the disgust object while the patient used his cognitive strategies during the down-regulation conditions.

#### Picture rating test

Repeated mesures two-way ANOVA analysis results showed no significant effect of time F(1, 29) = 0.530, p = 0.472, and condition F(1, 29) = 1.74, p = 0.198 on the arousal ratings. No interaction between time and condition was observed either F(1, 29) = 0.247, p = 0.623.

No significant effect of time F(1, 29) = 0.02, p = 0.89 and the condition F(1, 29) = 2.385, p = 0.133 was observed on the valence ratings too. Also, the interaction between time and the condition was not significant F(1, 29) = 1.256, p = 0.272.

Effect of time F(1, 29) = 2.201, p = 0.149 and condition F(1, 29) = 2.325, p = 0.138 on the symptom provocation ratings were found non-significant. There was no significant interaction between the time and the condition for the symptom provocation ratings F(1, 29) = 1.293, p = 0.265.

Although no time, condition and time x condition interaction effect were observed on arousal, valence and the symptom provocation ratings, paired samples t-tests revealed significant differences on valence and the symptom provacation ratings between the baseline and down-regulation conditions in the post-test. The patient rated the pictures as less symptom provocative and having less negative valence in the post-test down-regulation conditions (Valence baseline M = 1884.5, std = 95.76, down-regulation M = 1825.33, std = 166.06, p = 0.05; sypmtom provacation baseline M = 1708.07, std = 232.47, down-regulation M = 1633.53, std = 240.25, p<0.05).

#### Physiological measures during picture ratings (SCL & HR)

We did not observe a statistically significant difference in the SCL between baseline (M = 1.43, sd = 0.18) and the down-regulation (M = 1.43, sd = 0.22) conditions in the pre-test. The difference between the conditions for the SCL in the post-test was not significant either (Baseline M = 1.39, sd = 0.31; Down-regulation M = 1.42, sd = 0.28).

Also, HR difference between baseline (M = 81.96, sd = 4.94) and the down-regulation (M = 81.77, sd = 2.42) in the pre-test and post-test did not show any significance (Baseline M = 84.92, sd = 1.89; Down-regulation M = 85.05, sd = 2.86).

## Discussion

Recent evidence suggest that OCD involving contamination obsessions and washing compulsions might be characterized by a disorder in disgust processing. Hyperactivity in the insula may play a particularly important role in mediating such putative disruptions [24, 25, 35]. In light of these findings, we designed a pilot study to explore the effects of rtfMRI-neurofeedback based anterior insula down-regulation on several behavioral, physiological and clinical measures of OCD patients with contamination fears and washing compulsions. Also, we aimed to examine the feasibility of our study procedures, validity of the tools, and overall approach for a larger study developing an rtfMRI-neurofeedback treatment approach for OCD. Yet, a further purpose was to introduce/present our methods, some of which were used for the first time (e.g., Ecological Disgust Test) in rtfMRI-neurofeedback studies.

GLM analysis show that down-regulation of the anterior insula is possible in OCD patients. In fact, all patients could decrease the BOLD signal with rtfMRI-neurofeedback in insula, albeit to different degrees, in the presence of disgust inducing stimuli. Although all patients showed increased activity in the anterior insula during the down-regulation condition on the first rtfMRI-neurofeedback training day, they showed a decrease in activity in the same ROI through the following sessions. In addition to the decreased activity in the anterior insula, an increased activity during the down-regulation conditions in the middle temporal gyrus and left postcentral gyrus was observed in all the patients.

Patients were less successful at down-regulation during the transfer runs in comparison to the the regular training runs. Hence, including more transfer runs in the experimental protocol would be crucial in future experiments, in order to improve the behavioral results measured outside the scanner environment. Some further steps which can be taken to improve the self-regulation performance are discussed below.

In operant conditioning, the success in learning a new behavior is determined by the contingency of the behavior-consequence associations [65]. In rtfMRI-neurofeedback experiments, the association between a mental strategy that successfully regulates a neural signal (behavior) and feedback (consequence) can be affected by the temporal delay of the feedback signal [63]. In the great majority of rtfMRI-neurofeedback studies, feedback is continuously presented with minimal delay, approximately 2s [50]. However, because of insula’s role in feeling, motivation and self-monitoring [21, 66], we anticipated that there could be potential activity increases in the insula due to reward processing when the feedback is presented in real-time. To prevent such an increase in insula activity in opposition to the desired decrease of this study, we followed a different approach of providing delayed feedback [67, 68]. According to this method, BOLD amplitudes in the anterior insula during the down-regulation block was averaged, and a proportional level of monetary reward was computed and presented to the patient at the end of each down-regulation block instead of presenting the reward values in real-time. However, some studies have suggested that uncoupling the cognitive task from the feedback by providing the feedback at shorter intervals might be a better way to achieve a particular behavior–consequence association when mental imagery is used during self-regulation [69, 70]. In line with the above suggestion, with an objective to improve the contingency of the feedback, in our followup study it might be useful to provide patients information about their ongoing brain activity at 10 s intervals (three times throughout each down-regulation block). To avoid cognitive or visual distractions, which can be caused by the presence of the graphical thermometer next to the disgust inducing pictures in the screen, it would be beneficial to locate each picture inside a frame which can provide neurofeedback, for example by color changes. In this manner, it would be possible to separate the neurofeedback information, which is presented more often from the reward, which will be presented only at the end of each block.

As an alternate approach to feedback computation, it might also be interesting to consider the “hits” approach (currently used only as an offline analytical approach) for the feedback calculation in the follow-up studies in combination with an intermittent feedback to gain sensitivity to trial-by-trial variations.

In addition, the target regions selected for self-regulation and the direction of regulation (either up or down) are crucial aspects in brain self-regulation experiments. The majority of previous rtfMRI-neurofeedback studies used single ROI up-regulation paradigms in which participants were been trained to increase activity of one target region [48–50]. However, relatively fewer rtfMRI studies showed that subjects could learn to down-regulate the brain activity e.g., in amygdala [71], subgenual anterior cingulate [72], auditory cortex [73] and anterior cingulate cortex [74]. Decreasing the BOLD signal might be harder as compared to increasing it [41]. Our preliminary results are important in showing the possibility of down-regulation of an abnormally activated brain region in psychiatric populations.

Considering that OCD affects a distributed fronto-striatal circuit, a connectivity-based or a pattern classifier-based neurofeedback study design, instead of a single ROI design, might be an interesting possibility in future experiments. In a recent study, Weygandt and colleagues [75] investigated whether fear, disgust and neutral emotional states can be decoded from brain patterns of fMRI information in OCD patients and healthy controls. Their results indicated that fMRI data contains information about OCD-relevant fear stimuli, and using this information it is possible to distinguish between OCD patients and healthy controls. These results suggest that future studies could also investigate the use of classifier-based neurofeedback for the treatment of OCD.

Regarding the behavioral effects due to rtfMRI-neurofeedback training, it is not possible to make clear conclusions from this pilot study with only 3 patients. However, in two patients (Patient 1 and Patient 3) positive changes in the behavioural tests were observed. The aforementioned patients improved their capability to bear the physical proximity of a real-world, disgusting, symptom-provoking object, by employing their succesful cognitive strategies for down-regulation after the training. These results are forward-looking and trend-setting for the further development rtfMRI-neurofeedback, in light of previous studies that were limited to testing the immediate effects of self-regulation on behavior and symptoms by conducting tests inside the scanner environment [40, 44, 46, 76]. Novel symptom-specific tests, like EDT, would represent significant progress in the field for exploring the transfer and application of self-regulation skills to real-world situations.

It is important to stress that as both down-regulation and baseline conditions were interspersed in the same runs (both in the pre- and post-training test), the differences in behavioral/physiological measurements between baseline and down-regulation can not be attributed to habituation, effect of time, or general therapy that patients underwent, as those would have influenced both conditions. However, although all the stimuli presented in the pre- and the post behavioral tests were the same, there was an asymmetry in the pre- and the post-test instructions. We had assumed that there would not be differences between the baseline and the down-regulation conditions in the pre-tests but that there would be differences between these conditions in the post-test depending on the success in learning self-regulation. In accordance with the above assumption, we did not instruct the patients to use any cognitive strategy in the pre-test. Further, we supposed that this would allow us to obseerve the baseline behavioral and physiological values before the training. However, it would have been better if we had instructed the patients exactly the same way in both the pre- and post-tests as it would have allowed us to observe the instructions on brain activity during the pre-test. This potential limitation of the present study should be taken into account in future study designs.

In parallel with the EDT results, the same two patients also showed improvements in the picture rating test in two dimensions, i.e., valence and OCD symptom provocation. During the post-tests, patients rated the disgust evoking pictures with less negative valence, and as less symptom provocative following down-regulation blocks.

Patient 2, did not display any improvement in these dimensions. In fact, the patient's Y-BOCs scores were higher at the end of the experiments. Because the questionnaires used as clinical measures, are self-administered, and because we do not ask patients to use their mental strategies during the administration of the questionnaires, it is not possible to be certain whether the positive or negative changes in the scores could be attributed to the rtfMRI-neurofeedback training, or to other factors active during this period.

Because the main purpose of this study was to see the feasibility of the method and optimization of the measurements for a controlled, future study, we wanted to keep the heterogeneity of the patients to figure out the possible effects of the rtfMRI-neurofeedback training on different patient subgroups. In light of Patient-2’s lack of behavioral improvement and relatively low Y-BOCs scores at the beginning of the measurements, in future studies it might be useful to define a threshold for the clinical symptomatology (measured by Y-BOCs) for selecting only the patients whose scores are above that threshold.

Non-consistent results were observed for HR measures collected inside the scanner. Two patients (patient 1 and patient 2), displayed decreased heart rates during the down-regulation conditions. However, patient 3 had higher heart rates while down-regulating through the runs. HR measures, recorded while the patients were looking at the disgust inducing pictures outside the scanner, did not reveal a significant difference between the baseline and the down-regulation conditions, in the post-tests, in all three patients.

Previous psycho-physiological investigations exploring responses towards disgust inducing stimuli are limited in number and provide conflicting results, including heart rate. Although some studies show elevated HR in the conditions when healthy subjects are presented with symptom provoking pictures [77–81], others point to a deceleration in the HR as an indicator of parasympathetic activity [82–84]. Studies that have measured OCD patients’ physiological responses towards disgust inducing stimuli reported contradictory results as well. While some experimenters found elevated autonomic nervous system activity and HR [85–88], other studies reported HR decelerations which is interpreted as a sign of orientation and not avoidance or stimulus rejection [89–92]. One of the reasons for these conflicting findings might be the type of the stimuli used in the experiments. While the disgust emotion elicited in relation to contamination and pollution (e.g., pictures of dirty toilets, cockroaches, maggots on food, foul smells, facial expressions of expelling food), is characterized by HR acceleration, disgust elicited in relation to mutilation, injury and blood (e.g., injections, mutilation scenes, bloody injuries), seems to be characterized by a pattern of HR deceleration [93]. In our set of stimuli we included both contamination and mutilation types of images. Therefore, and depending on the heterogeneity of the OCD patients, using both kind of pictures together in the same measurement might cause a neutralizing effect in HR measures. The differences on anxiety levels in our small group of patients might have also contributed to this inconsistency in HR results. Patient 3, who showed elevated HR during the down-regulation conditions in the scanner, showed also the highest anxiety level (both in trait and state-anxiety measures). Patients' elevated HR during down-regulation conditions might also be attributed to performance anxiety and reward expectancies.

Disgust is consistently reported to be associated with increased electrodermal activity [93]. However, we did not observe a significant difference between disgust provoking and neutral pictures and skin conductance level levels. There were no significant differences in levels between baseline and down-regulation blocks while patients were viewing disgust inducing pictures either. Because we measured the mean skin conductance levels throughout baseline and the down-regulation blocks, these results might be due to the presentation of mixed type of disgust inducing pictures.

Subjective reports of patients at the end of the rtfMRI-neurofeedback training periods indicate that patients used reappraisal or suppression strategies to gain control over the feedback signal. With continued training, subjects modified their strategy to maximize their feedback and reward. Because different emotional regulation mechanisms may have different effects on physiological responses such categorization could partly explain the heterogenity in the heart rate and the skin conductance level measurements as well.

Finally, an important question as to whether the BOLD up- or down-regulation directly leads to the physiological and clinical changes observed in the patients, or the invoking of the appropriate mental imagery and cognitive strategies leads to those physiological changes, or both mechanisms are interwoven remains unanswered in this or any of the rtfMRI-neurofeedback studies so far. This is a topic of ongoing and future research.

In summary, in this pilot study we investigated the application of rtfMRI-neurofeedback for the down-regulation of BOLD activity in the anterior insula in OCD patients. We also explored the feasibility of several behavioral and physiological pre- and post-training tests and clinical assessments that could be used in a future long-term study. Our results indicate that with sufficient training OCD patients can down-regulate the BOLD activity in their anterior insula in the presence of disgust inducing stimuli. The two tests that were designed to examine the effect of this self-control in real-world conditions outside the scanner (picture rating tests and the EDT) have important implications for future studies.

To improve the consistency of self-regulation, it might be crucial to combine rtfMRI- neurofeedback sessions with extended neurofeedback training outside the scanner using portable EEG and/or functional near infrared spectroscopy (fNIRS). For this purpose, it would be useful to identify the EEG and/or fNIRS correlates of down-regulation of the BOLD signal in the anterior insula in a patient-specific manner.
