# Correction: Self-Regulation of Anterior Insula with Real-Time fMRI and Its Behavioral Effects in Obsessive-Compulsive Disorder: A Feasibility Study

**DOI:** 10.1371/journal.pone.0145027

**Published:** 2015-12-16

**Authors:** Korhan Buyukturkoglu, Hans Roettgers, Jens Sommer, Mohit Rana, Leonie Dietzsch, Ezgi Belkis Arikan, Ralf Veit, Rahim Malekshahi, Tilo Kircher, Niels Birbaumer, Ranganatha Sitaram, Sergio Ruiz

There is an affiliation error. The affiliation for the 11^th^ author is not indicated. Ranganatha Sitaram is not affiliated with #2 but instead is affiliated with #7: Institute of Medical and Biological Engineering, School of Engineering, and Department of Psychiatry and Section of Neuroscience, School of Medicine. Pontificia Universidad Catolica de Chile, Santiago, Chile.
